# Supplementary material for: Content-rich biological network constructed by mining PubMed abstracts
Source: BMC Bioinformatics. 2004 Oct 8;5:147. doi: 10.1186/1471-2105-5-147 (PMC528731; doi:10.1186/1471-2105-5-147)
Supplement: Additional File 2 — The original results of the above study (non-essential files are deleted to keep the file size under the limit set by BMC bioinformatics). [file 1471-2105-5-147-S2.bz2 › chilibotAdditionalFile2/dip05/108ID7559507E445/html/IGF1R_IRS1.html]

 


 **IGF1R** and **IRS1** 
  
Found 109 abstracts in PubMed, retrieved 05.  
 

 What does Google say? 
 PDF only 
| .edu only 

---

- J NutrJ Nutr,   **Conjugated Linoleic Acid Downregulates Insulin Like Growth Factor I Receptor **[IGF1R]** Levels in HT 29 Human Colon Cancer Cells.** .
  Conjugated linoleic acid CLA has chemoprotective properties in a variety of experimental cancer models.
  We have previously observed that dietary CLA inhibits colon tumorigenesis induced by dimethylhydrazine in rats.
  In addition, our in vitro studies have shown that CLA inhibits DNA synthesis and induces apoptosis in HT 29 cells, the human colon adenocarcinoma cell line.
  The insulin like growth factor IGF system regulates the growth of HT 29 cells by an autocrine mechanism.
  The present study examined whether the growth inhibitory effect of CLA is related to changes in the IGF system in HT 29 cells.
  To determine whether CLA inhibits IGF II production, HT 29 cells were incubated in serum free medium in the presence of various concentrations of CLA.
  CLA decreased protein levels of both mature and pro IGF II and IGF II transcripts.
  Whereas exogenous IGF I and IGF II produced an increase in cell number, neither IGF I nor IGF II counteracted the negative growth regulatory effect of CLA.
  Reverse transcriptase polymerase chain reaction and Western blot analysis of total cell lysates revealed that CLA decreased IGF I receptor IGF IR transcript and protein levels in a dose dependent manner.
  Immunoprecipitation Western blot studies revealed that CLA inhibited IGF I induced phosphorylation of IGF IR and insulin receptor substrate IRS 1 **[IRS1]**, recruitment of the p85 regulatory subunit of phosphatidylinositol 3 kinase PI3K to IGF IR, IGF IR associated PI3K activity, and phosphorylated Akt and extracellular signal regulated kinase ERK 1 2 levels.
  In conclusion, the inhibition of cell proliferation and induction of apoptosis by CLA in HT 29 cells may be mediated in part by its ability to decrease IGF II synthesis and to downregulate IGF IR signaling and the PI3K Akt and ERK 1 2 pathways.

  - Endocrinology< MedlineTA>Endocrinology,   **Cytokine Hormone Interactions Tumor Necrosis Factor alpha Impairs Biologic Activity and Downstream Activation Signals of the Insulin Like Growth Factor I Receptor **[IGF1R]** in Myoblasts.** .
    TNFalpha is elevated following damage to skeletal muscle.
    Here we provide evidence that TNFalpha acts on muscle cells to induce a state of IGF I receptor resistance.
    We establish that TNFalpha inhibits IGF I stimulated protein synthesis in primary porcine myoblasts.
    Similar results were observed in C 2 C 12 murine myoblasts, where as little as 0.01 ng ml TNFalpha significantly inhibits protein synthesis induced by IGF I.
    TNFalpha also impairs the ability of IGF I to induce expression of a key myogenic transcription factor, myogenin.
    The inhibition by TNFalpha of IGF I induced protein synthesis and expression of myogenin is not due to direct killing of myoblasts by TNFalpha.
    Although IGF I induces an approximately 19 fold induction in tyrosine phosphorylation of the beta chains of its receptor, TNFalpha does not inhibit this autophosphorylation.
    Instead, TNFalpha significantly reduces by approximately 50% IGF I stimulated tyrosine phosphorylation of two of the major downstream receptor docking molecules, insulin receptor substrate IRS 1 **[IRS1]** and IRS **[IRS1]** 2.
    These results establish that low picogram concentrations of TNFalpha acts on both porcine and murine myoblasts to impair tyrosine phosphorylation of both IRS 1 **[IRS1]** and IRS **[IRS1]** 2, but not the receptor itself.
    These data are consistent with the notion that very low physiological concentrations of TNFalpha interfere with both protein synthesis and muscle cell development by inducing a state of IGF I receptor resistance.

    - Cancer Res, 2003   Insulin like growth factors stimulate breast cancer proliferation, motility, and survival.The type I IGF receptor mediates the effects of IGF I.
      Thus, inhibition of **IGF1R** activation could inhibit IGF action in breast cancer cells.
      A single chain antibody directed against **IGF1R** has been shown to partially inhibit xenograft growth of MCF 7 cells in athymic mice.
      In this study, we have examined the effects of scFv Fc on **IGF1R** signaling in the estrogen receptor positive MCF 7 breast cancer cells in vitro and in vivo.
      The antibody stimulated **IGF1R** activation in vitro in MCF 7 cells and was unable to block IGF I effects.
      The antibody also stimulated proliferation of MCF 7 cells in monolayer growth assays.
      To determine how scFv Fc could stimulate in vitro growth yet inhibit in vivo tumor growth, we examined the effect of scFv Fc on **IGF1R** expression.
      In MCF 7 cells, scFv Fc down regulated **IGF1R** levels after 2 h, and the levels were greatly reduced after 24 h.
      In contrast, IGF I treatment over the same time period did not affect **IGF1R** levels.
      Twenty four h pretreatment of cells with scFv Fc blocked IGF I mediated phosphorylation of insulin receptor substrate 1 **[IRS1]** and subsequent extracellular signal regulated kinase 1 extracellular signal regulated kinase 2 and phosphatidylinositol 3 kinase activation.
      In contrast, cells treated with 5 nM IGF I for 24 h still retained the ability to further activate downstream signaling pathways in response to IGF I.
      Moreover, pretreatment of MCF 7 cells with scFv Fc rendered them refractory to further proliferation induced by additional IGF I.
      Twenty four h pretreatment of cells with scFv Fc also inhibited IGF I stimulated anchorage independent growth.
      scFv Fc did not enhance antibody dependent cell mediated cytotoxicity.
      In vivo, treatment of mice bearing MCF 7 xenograft tumors with scFv Fc resulted in near complete down regulation of **IGF1R**.
      Our data show that scFv Fc stimulates biochemical activation of **IGF1R**, then causes receptor down regulation, making MCF 7 cells refractory to additional IGF I exposure.
      These results indicate that such chimeric single chain antibodies against **IGF1R** have future potential in breast cancer therapy by causing down regulation of receptor.

      - Oncogene, 2003   **An inhibitory function for JNK in the regulation of IGF I signaling in breast cancer.** .
        Insulin like growth factor I receptor **[IGF1R]** IGF IR is frequently overexpressed in a variety of cancer types.
        Since many breast tumors and cancer cell lines overexpress IGF IR, we tested IGF I effects on chemotherapy treated breast cancer cells.
        IGF I protects from chemotherapy induced apoptosis, suggesting that overlapping signaling pathways modulate IGF I and chemotherapy treatment outcomes.
        Taxol and other chemotherapy drugs induce c Jun N terminal kinase JNK , a kinase that conveys cellular stress and death signals.
        Notably, in this paper we show that IGF I alone induces a potent JNK response and this activity is reversed by inhibition of phosphatidylinositol 3 kinase PI 3 kinase with LY294002 in MCF 7 but not T47D cells.
        Cotreatment of cells with chemotherapy and IGF I leads to additive JNK responses.
        Using cells overexpressing Akt, we confirm that IGF I mediated survival is Akt dependent.
        In contrast, overexpression of JNK significantly enhances Taxol induced apoptosis and inhibits IGF I survival effects.
        Further, JNK attenuates anchorage independent growth of MCF 7 cells.
        The inhibitory effect of JNK appears to be mediated by serine phosphorylation of IRS 1 **[IRS1]** insulin receptor substrate since both Taxol and IGF I treatment enhanced Ser 312 IRS 1 **[IRS1]** phosphorylation, while LY294002 blocked IGF I mediated phosphorylation.
        Taken together, these data provide a mechanism whereby stress or growth factors activate JNK to reduce proliferation and or survival in breast cancer cells.

        - Mol Endocrinol< MedlineTA>Mol Endocrinol,   The insulin receptor substrate 1 **[IRS1]** can translocate to the nuclei and nucleoli of several types of cells.
          Nuclear translocation can be induced by an activated insulin like growth factor 1 receptor **[IGF1R]** , and by certain oncogenes, like the SV40 T antigen and v src.
          We have asked whether IRS **[IRS1]** 2 could also translocate to the nuclei.
          In addition, we have studied the effects of functional mutations in the IGF IR on nuclear translocation of IRS **[IRS1]** proteins.
          IRS **[IRS1]** 2 translocates to the nuclei of mouse embryo fibroblasts expressing the IGF IR, but, at variance with IRS 1 **[IRS1]**, does not translocate in cells expressing the SV40 T antigen.
          Mutations in the tyrosine kinase domain of the IGF IR abrogate translocation of the IRS **[IRS1]** proteins.
          Other mutations in the IGF IR, that do not interfere with its mitogenicity but inhibit its transforming capacity, result in a decrease in translocation, especially to the nucleoli.
          Nuclear IRS 1 **[IRS1]** and IRS **[IRS1]** 2 interact with the Upstream Binding Factor , which is a key regulator of RNA polymerase I activity and, therefore, ribosomal RNA synthesis.
          In 32D cells, wild type, but not mutant IRS 1 **[IRS1]**, causes a significant activation of the rDNA promoter.
          The interaction of nuclear IRS **[IRS1]** proteins with UBF1 constitutes the first direct link of these proteins with the rDNA transcription machinery.
